# Comparative Clinical Evaluation of Resin-based Pit and Fissure Sealant and Self-adhering Flowable Composite: An *In Vivo* Study

**DOI:** 10.5005/jp-journals-10005-1552

**Published:** 2018-10-01

**Authors:** Saakshe Wadhwa, Ullal A Nayak, Damodhar Kappadi, Deepesh Prajapati, Reena Sharma, Apurva Pawar

**Affiliations:** 1 Postgraduate Student, Department of Pedodontics and Preventive Dentistry, NIMS Dental College, Jaipur, Rajasthan, India; 2 Professor and Head, Department of Pediatric Dentistry, IBN Sina National College for Medical Science, Jeddah, Kingdom of Saudi Arabia; 3 Reader, Department of Pedodontics and Preventive Dentistry, NIMS Dental College, Jaipur, Rajasthan, India; 4 Senior Lecturer, Department of Pedodontics and Preventive Dentistry, NIMS Dental College, Jaipur, Rajasthan, India; 5 Postgraduate Student, Department of Pedodontics and Preventive Dentistry, NIMS Dental College, Jaipur, Rajasthan, India; 6 Postgraduate Student, Department of Pedodontics and Preventive Dentistry, NIMS Dental College, Jaipur, Rajasthan, India

**Keywords:** Marginal integrity, Pit and fissure sealant, Retention, Self-adhering flowable composite.

## Abstract

**Aim:**

The study evaluated the use of self-adhering flowable composite as a fissure sealant and compared it with a resin-based pit and fissure sealant.

**Materials and methods:**

Forty children were selected for the study and all their four permanent first molars were subjected to fluoride free pumice oral prophylaxis. Their occlusal fissures were then prepared with fissurotomy bur using high-speed handpiece under cotton roll isolation and low volume suction. Simple random sampling was done and accordingly a child either received either a resin-based fissure sealant or a self-adhering flowable composite on the prepared fissure. All the restorations were clinically evaluated using Ryge’s direct evaluation criteria for four times i.e., immediately after the treatment, at the end of 3rd, 6th and 12th month. The retention was evaluated using Horowytz criteria.

**Results:**

The retention rate of Dyad flow after one year was significantly higher than that of Helioseal-F (p = 0.015). The marginal integrity of Dyad Flow was significantly better than that of Helioseal-F during every evaluation period (p < 0.05). Both retention and marginal integrity of both sealants were similar in maxillary and mandibular molars at all evaluation periods.

**Conclusion:**

Dyad flow can be used as an alternative to the conventional fissure sealant.

**Clinical significance:**

In pediatric dentistry, where shorter appointment time is warranted, the self-adhering composite has the edge over conventional fissure sealant.

**How to cite this article:** Wadhwa S, Nayak UA, Kappadi D, Prajapati D, Sharma R, Pawar A. Comparative Clinical Evaluation of Resin-based Pit and Fissure Sealant and Self-adhering Flowable Composite: An *In-vivo* Study. Int J Clin Pediatr Dent. 2018;11(5):430-434.

## INTRODUCTION

The pits and fissures of teeth have a high predilection for dental caries.^1^ The sealant placement has been proved to be a cost-effective and reliable method of preventing fissure caries in children. Longer the sealant remains intact, and lesser is the incidence of recurrent caries beneath it.^2^ The properties of sealants can be enhanced by adding filler particles, fluoride, and color to the resin material. Silane-treated amorphous silica of particle size 0.016 micrometers is added as filler, and one such sealant is Helioseal-F.^3^

The self-adhering flowable composites are the result of recent advances in restorative dentistry, with increased flowability, higher retention rates,^4^ and shorter chair-side time which are of advantage while restoring a child’s tooth.^5^ Dyad flow is one such material manufactured by Kerr, USA, which synergizes the technology of composite resins and bonding agent, i.e., acidic adhesive monomer into the flow-able composite itself. It gains retention with tooth structure by chemical and/or micromechanical means.

Studies comparing sealants and flowable composite are based on single criteria, but none have conclusively dealt with the comparison between the gold standard sealant and self-adhering composite based upon retention and integrity of restorative margins which form the backbone of caries prevention among children in early mixed dentition.

Owing to the novelty of this material, the present study was aimed to evaluate the retention and marginal integrity of self-adhering flowable composite used as a fissure sealant and to compare it with a resin-based pit and fissure sealant.

## MATERIALS AND METHODS

Forty children, aged 7 to 10 years, who were attending our outpatient department, were randomly selected for the study based on the following inclusion criteria.

### Inclusion Criteria

- Age group of 7 to 10 years
- All four first permanent molars that have completely erupted with fissures either intact, sound or retentive which are stained or calcified but not carious
- Subjects who were not undergoing any other preventive dental health programme
- Normal healthy children
- Availability for the duration of the study
- Satisfactory dental care performed at home
- The willingness of the patient to accept the treatment.

### Exclusion Criteria

- Special child, a child with compromised systemic health
- Long-term medication affecting the salivary flow
- Children enrolled for other studies or fluoridation program
- Adverse reaction reported to any dental material
- Uncooperative child.

The children selected based upon above-mentioned criteria were then randomly divided into two equal groups (odd and even) for the evaluation of the sealants.

*Group I:* Resin based pit and fissure sealant - Helioseal-F [80 teeth]

*Group II:* Self-adhering flowable composite-Dyad Flow (80 teeth)

The parent’s or guardian’s informed consent and the ethical clearance certificate from the University were obtained before the onset of the study.

Firstly, fluoride free pumice oral prophylaxis was performed. Occlusal fissures were prepared with Micro STF fissurotomy bur under cotton roll isolation and low volume suction.

*Group I:* Helioseal-F sealant group: The prepared teeth were subjected to etching, washing, and drying as per instructions of use. The adhesive and Helioseal-F sealant were consecutively applied and light cured.

*Group II:* Dyad flow self-adhering flowable composite group: The prepared teeth were subjected to etching, washing, and drying as per instructions of use. The adhesive and Dyad flow were consecutively applied and light cured.

The satisfactory, marginal seal between the material and the tooth surface was confirmed using a probe. A carbon marker was used to evaluate occlusion and premature contacts if any were removed accordingly.

All the cases were clinically evaluated four times i.e., immediately after the treatment, and at the end of 3rd, 6th and 12th month based on Ryge’s direct evaluation criteria.^6^ The retention was evaluated based on Horowytz criteria.^7^

The values obtained were recorded in Microsoft Excel and subjected to statistical analysis for the comparison between the two groups.

## RESULTS

**Graph 1:**
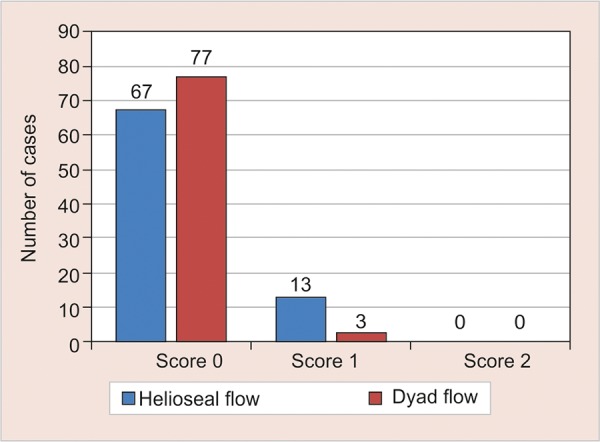
Comparative evaluation of retention of Helioseal-F and Dyad flow at different evaluation periods and between upper and lower permanent molars

Table 1 and Graph 1 describe the retention of Helioseal-F and Dyad flow at different periods, and between upper and lower molars, there was no statistical significance between the Helioseal-F and Dyad Flow at either 3 or 6 months evaluation period. However, the retention rate of Dyad Flow was significantly higher than Helioseal-F (p = 0.015) at one year evaluation period. The retention rates of both sealants were similar in maxillary and mandibular molars at all evaluation periods (p > 0.05).

**Graph 2:**
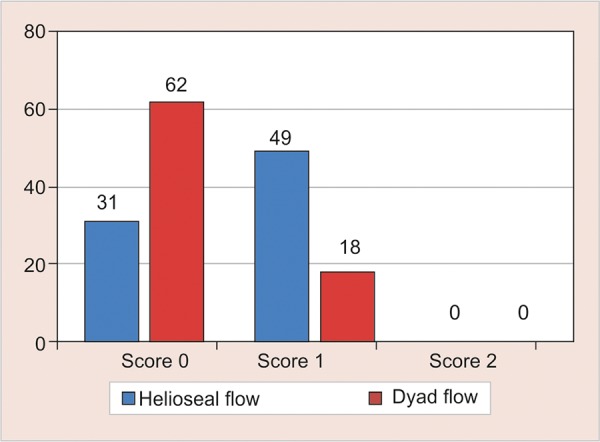
Comparative evaluation of marginal integrity of Helioseal-F and Dyad flow at different evaluation periods and between upper and lower permanent molars

Table 2 and Graph 2 compared the marginal integrity of Helioseal-F and Dyad flow at different evaluation periods between upper and lower molars. The marginal integrity of Dyad flow was found to be significantly better than Helioseal-F at all evaluation periods (p < 0.05). The marginal integrity of both sealants was similar in maxillary and mandibular molars at all evaluation periods.

**Table 1:** **Table 1:** Comparative evaluation of retention of Helioseal-F and Dyad Flow at different evaluation periods and between upper and lower permanent molars

		*Retention of sealants*																											
		*Helioseal F N = 80*												*Dyad flow N = 80*															
		*Score 0*				*Score 1*				*Score 2*				*Score 0*				*Score 1*				*Score 2*							
*Evaluation period*		*No.*		*%*		*No.*		*%*		*No.*		*%*		*No.*		*%*		*No.*		*%*		*No.*		*%*		*Chi square value*		*p-value*	
Immediate		80		100		0		0		0		0		80		100		0		0		0		0		–		–	
3 months		76		95		4		5		0		0		78		96.3		2		3.7		0		0		0.693		0.681	
6 months		74		92.5		6		7.5		0		0		77		94.4		3		5.6		0		0		1.060		0.495	
12 months		67		83.8		13		16.2		0		0		77		94.4		3		5.6		0		0		6.944		0.015	
		*Retention of Helioseal-F*																											
		*Maxillary teeth*												*Dyad flow N = 80*																	
		*Score 0*				*Score 1*				*Score 2*				*Score 0*				*Score 1*				*Score 2*							
*Evaluation period*		*No.*		%		*No.*		*%*		*No.*		*%*		*No.*		*%*		*No.*		*%*		*No.*		*%*		*Chi square value*		*p-value*	
Immediate		40		100		0		0		0		0		40		100		0		0		0		0		–		–	
3 months		37		92.5		3		7.5		0		0		39		97.5		1		2.5		0		0		1.053		0.615	
6 months		36		90		4		10		0		0		38		95		2		5		0		0		0.721		0.675	
12 months		33		82.5		7		17.5		0		0		34		85		6		15		0		0		0.092		1.000	
		*Retention of Dyad flow*																											
		*Maxillary teeth*												*Dyad flow N = 80*																	
		*Score 0*				*Score 1*				*Score 2*				*Score 0*				*Score 1*				*Score 2*							
*Evaluation period*		*No.*		%		*No.*		*%*		*No.*		*%*		*No.*		*%*		*No.*		*%*		*No.*		*%*		*Chi square value*		*p-value*	
3 months		39		97.5		1		2.5		0		0		39		97.5		1		2.5		0		0		0.000		1.000	
6 months		38		95		2		5		0		0		39		97.5		1		2.5		0		0		0.346		1.000	
12 months		38		95		2		5		0		0		39		97.5		1		2.5		0		0		0.346		1.000	

p ≤ 0.05 is significant

## DISCUSSION

The initial cost of preventive measures like sealants are estimated to be higher than that of restorative materials, but in the long term they prove to be more cost-effective as the tooth would be maintained in a state of health.^8^

After polymerization, Helioseal-F leaves behind a smooth surface and entraps fewer or no air bubbles and needs minimal time for finishing.^8^

The Dyad flow contains pre-polymerized filler particles which have better polishability, mechanical properties, ease of handling and flow, thus allowing deeper penetration into the fissures.^4,8^ Hence, the present study was aimed to assess its efficacy as an alternative to fissure sealant.

In the present study at the end of 3rd month, 95% of resin-based sealants and 96.3% of self-adhering flowable composites were intact. However, at the end of 6th month, 92.2% of resin-based sealant and 94.4% self-adhering flowable composite were intact. The retention of Heli-oseal F was comparable to certain other studies.^9,10^ The high retention rate for self-adhering flowable composite observed in this study could be related to its ease of application, good flow, less air bubble incorporation and increased working time.

The low retention rate of Helioseal-F is related to calcium fluoride which is formed rapidly thereby reducing its sealing to enamel surface. The presence of fillers makes its viscosity higher by decreasing its penetrability.^11^ However, the increased retention observed in this study could be related to the use of an adhesive which increases its cost and time.^12^

Adequate curing of the material is important for success. The curing light should be tested monthly to make certain that intensity is optimal. Accidental contamination of saliva, particularly when treating newly erupted permanent molars in young children could partially explain the reason for poor retention rates in a few studies involving young children.^13,14^

The isolation performed by a cotton roll or rubber dam exhibited no significant differences when sealant retention was evaluated.^15^ Hence, in the present study, cotton roll isolation opted. However, it is reported that the mandibular molars needed retreatment more often than maxillary molars. This may be due to newly erupting mandibular permanent molar, the distal tissue flap or operculum of which seemed to be present for a longer period thereby making isolation of the occlusal surface more difficult.^15^

**Table 2:** **Table 2:** Comparative evaluation of Marginal Integrity of Helioseal-F and Dyad flow at different evaluation periods and between upper and lower permanent molars

		*Marginal Integrity of sealants*																											
		*Helioseal F N = 80*												*Dyad flow N = 80*															
		*Score 0*				*Score 1*				*Score 2*				*Score 0*				*Score 1*				*Score 2*							
*Evaluation period*		*No.*		*%*		*No.*		*%*		*No.*		*%*		*No.*		*%*		*No.*		*%*		*No.*		*%*		*Chi square value*		*p-value*	
Immediate		80		100		0		0		0		0		80		100		0		0		0		0		–		–	
3 months		61		76.3		19		23.7		0		0		75		93.8		5		6.2		0		0		9.608		0.003	
6 months		47		58.8		33		41.2		0		0		69		86.3		11		13.7		0		0		15.172		0.000	
12 months		31		38.8		49		61.2		0		0		62		77.5		18		22.5		0		0		24.677		0.000	
		*Marginal Integrity of Helioseal-F*																											
		*Maxillary teeth*												*Dyad flow N = 80*															
		*Score 0*				*Score 1*				*Score 2*				*Score 0*				*Score 1*				*Score 2*							
*Evaluation period*		*No.*		*%*		*No.*		*%*		*No.*		*%*		*No.*		*%*		*No.*		*%*		*No.*		*%*		*Chi square value*		*p-value*	
Immediate		40		100		0		0		0		0		40		100		0		0		0		0		–		–	
3 months		34		85		6		15		0		0		27		67.5		13		32.5		0		0		3.382		0.114	
6 months		26		65		14		35		0		0		21		52.5		19		47.5		0		0		1.289		0.364	
12 months		16		40		24		60		0		0		15		37.5		25		62.5		0		0		0.053		1.000	
		*Marginal Integrity of Dyad flow*																											
		*Maxillary teeth*												*Dyad flow N = 80*															
		*Score 0*				*Score 1*				*Score 2*				*Score 0*				*Score 1*				*Score 2*							
*Evaluation period*		*No.*		*%*		*No.*		*%*		*No.*		*%*		*No.*		*%*		*No.*		*%*		*No.*		*%*		*Chi square value*		*p-value*	
Immediate		40		100		0		0		0		0		40		100		0		0		0		0		–		–	
3 months		40		100		0		0		0		0		35		87.5		5		12.5		0		0		5.333		0.055	
6 months		37		92.5		3		7.5		0		0		32		80		8		20		0		0		2.635		0.193	
12 months		31		77.5		9		22.5		0		0		31		77.5		9		22.5		0		0		0.000		1.000	

p ≤ 0.05 is significant

The use of a hybrid material as sealants with prior acid etching of the enamel enabled the sealant to permanently act as a physical barrier with the anti-cariogenic effect provided by the material. The use of same technique resulted in excellent retention rates for Helioseal-F in this study. ^16^

The adhesion of the resin to the enamel depends not only on the application of acid etch to the enamel but also on other factors like polishing of the dental surface prior to etching, etching time, the concentration of the acid and the type of acid used.^17^ Regarding etching time, many authors have already advocated a reduction of time since they observed no difference in the adhesion of sealants and they all recommended a standard time of 15 to 20 seconds.^9,18^ As per this recommended time, we have used a standard time of 20 seconds for each tooth before sealant placement.

A thin film of plaque retained on the fissure can jeopardize the bond between sealant and enamel. Thus, an invasive technique, which opens up the pits and fissures, cleans and widens the narrow fissures with a small fissurotomy bur, may easily reveal any caries in the fissure that could go unnoticed otherwise. Mechanical preparation of the occlusal surface may offer superior fissure sealant retention in everyday procedures where cotton roll isolation is used.^19^ In the present study, mechanical preparation would have resulted in higher retention.

The resin sealant success depends on its retention as well as integrity.^20^ The resin sealant’s ability to halt the initiation of fissure caries is restricted to the formation of a physical barrier, which blocks any metabolic exchange possible between the cariogenic microorganisms in the fissure and the surrounding oral environment.^20,21^ Several authors have also proved that the caries incidence is low when there is full retention of the sealant.^22,23^

For the marginal integrity, fissure sealant success rates reported in the literature were 98.2% and 95.5%, respectively.^5^ In the present study, the marginal integrity of resin-based sealant and the self-adhering flowable composite was well maintained in 76.3% and 93.8% respectively at the end of 3rd month. However, at the end of 6th month, 58.8% of resin-based sealant and 86.3% of self-adhering flowable composite showed good marginal integrity.

As the Dyad Flow has been recently introduced, there are not many studies to support our result. The present study provides some data to encourage further research into the use of Dyad flow as an alternate to sealant use in pediatric dentistry.

Improper marginal sealing exhibited by sealants can cause the bacteria and its fluids to penetrate the sealant-tooth margin, thereby increasing the incidence of recurrent caries beneath the sealant.^24^ Hence, for the long-term success of pit and fissure sealants, retention and proper adhesion to enamel surface is mandatory.

## CONCLUSION

At one year evaluation period, Dyad flow exhibited significantly better retention than that of Helioseal-F.

The marginal integrity of Dyad flow was significantly better than that of Helioseal-F at either 3, 6, 12 months evaluation period.

Both in the maxillary and mandibular molars, the marginal integrity of Dyad flow was significantly superior.

Thus, Dyad flow can opt as an alternate to fissure sealants. However, further long-term *in vivo* research may be necessary evaluating other material properties to validate its use as a suitable sealant alternative.

## References

[1] Salama FS, AL-Hammad NS (2002). Marginal seal of sealant and compomer materials with and without enameloplasty. Int J Paediatr Dent.

[2] Simonsen RJ (1978). Chapter 2: Pit and fissure sealants. Clinical Applications of the Acid Etch Technique..

[3] Sanders BJ, Henderson HZ, Avery DR, Mc Donald RE, Avery DR (2004). Pit and Fissure Sealants and Preventive Resin Restorations.. Dentistry for the child and Adolescent,.

[4] Lele GS, Bhide PC (2016). Evaluation of Dyad Flow as a Pit and Fissure Sealant: An In-Vitro Pilot Study. Int J Oral Health Med Res.

[5] Deshpande A, Sudani U, Bargale S, Poonacha KS, Kadam M, Joshi N (2016). Six months clinical performance of self etch-self adhesive flowable composite and conventional pit-and-fissure sealants in 7 to 10 year old children. J Adv Med Dent Sci Res..

[6] Ryge G, Snyder M (1973). Evaluating the clinical quality of restorations. J Am Dent Assoc.

[7] Horowitz HS, Heifetz SB, Poulsen S (1976). Adhesive sealant clinical trial: an overview of results after four years in Kalispell, Montana. The Journal of preventive dentistry..

[8] Ninawe N, Ullal NA, Khandelwal V (2012). A 1-year clinical evaluation of fissure sealants on permanent first molars. Contemporary clinical dentistry..

[9] Sol E, Espasa E, Boj JR, Canalda C (2000). Effect of different prophylaxis methods on sealant adhesion. The Journal of clinical pediatric dentistry..

[10] Ibsen RL (1973). Use of a filled diacrylate as fissure sealant: one-year clinical study. The Journal of the American Society for Preventive Dentistry..

[11] Yildiz E, Dorter C, Efes B, Koray F (2004). A comparative study of two fissure sealants: a 2-year clinical follow-up. Journal of oral rehabilitation..

[12] Feigal RJ, Quelhas I (2003). Clinical trial of a self-etching adhesive for sealant application: success at 24 months with Prompt L-Pop. American journal of dentistry..

[13] Burt BA, Herman DS, Silverstone LM (1977). Sealant retention and effects on occlusal caries after 2 years in a public program. Community dentistry and oral epidemiology..

[14] Stephen KW, Sutherland DA, Trainer J (1976). Fissure sealing by practitioners. First year retention data in Scottish 6-year-old children. British dental journal..

[15] Dennison JB, Straffon LH, More FG (1990). Evaluating tooth eruption on sealant efficacy. The Journal of the American Dental Association..

[16] Simonsen RJ (2002). Pit and fissure sealant: review of the literature. Pediatric dentistry..

[17] Autio-Gold JT (2002). Clinical evaluation of a medium-filled flowable restorative material as a pit and fissure sealant. Operative dentistry..

[18] Duggal MS, Tahmassebi JF, Toumba KJ, Mavromati C (1997). The effect of etching times on the retention of fissure sealants in primary and first permanent molars. International journal of paediatric dentistry..

[19] Blackwood JA, Dilley DC, Roberts MW, Swift EJ Jr. (2002). Evaluation of pumice, fissure enameloplasty and air abrasion on sealant microleakage. Pediatric dentistry.

[20] Going RE, Conti AJ, Haugh LD, Grainger DA (1976). Two-year clinical evaluation of a pit and fissure sealant. Part II: Caries initiation and progression.. The Journal of the American Dental Association [Internet]. Elsevier BV;.

[21] Handelman SI, Washburn F, Wopperer P (1976). Two year report of sealant effect on bacteria in dental caries. The Journal of the American Dental Association..

[22] Poulsen S, Breiruti N, Sadat N (2001). A comparison of retention and the effect on caries of fissure sealing with glass-ionomer and resin-based sealant. Community Dent Oral Epidemiol.

[23] Fuks AB, Grajower R, Shapira J (1984). In vitro assessment of marginal leakage of sealants placed in permanent molars with different etching times. ASDC journal of dentistry for children..

[24] Pardi V, Sinhoreti MA, Pereira AC, Ambrosano GM, Meneghim MD (2006). In vitro evaluation of microleakage of different materials used as pit-and-fissure sealants. Brazilian dental journal..

